# The Association between Psychological and Behavioral Economic Factors and the Rapid Assessment Disuse Index (RADI) during the COVID-19 Pandemic

**DOI:** 10.3390/ijerph21081040

**Published:** 2024-08-07

**Authors:** Clare Meernik, Qing Li, Jeffrey Drope, Ce Shang, Tammy Leonard, Bob M. Fennis, Mahmoud Qadan, Carolyn E. Barlow, Laura F. DeFina, Reid Oetjen, Loretta DiPietro, Kerem Shuval

**Affiliations:** 1 Department of Research, The Cooper Institute, Dallas, TX 75230, USA; 2 Bloomberg School of Public Health, Johns Hopkins University, Baltimore, MD 21205, USA; 3 College of Medicine, The Ohio State University, Columbus, OH 43210, USA; 4 Peter O’Donnell Jr. School of Public Health, University of Texas Southwestern Medical Center, Dallas, TX 75390, USA; 5 Harold C. Simmons Comprehensive Cancer Center: University of Texas Southwestern Medical Center, Dallas, TX 75235, USA; 6 Faculty of Economics and Business, University of Groningen, 9747 Groningen, The Netherlands; 7 School of Business Administration, University of Haifa, Haifa 3103301, Israel; 8 School of Global Health Management and Informatics, College of Community Innovation and Education, University of Central Florida, Orlando, FL 32816, USA; 9 Department of Exercise and Nutrition Sciences, Milken Institute School of Public Health, The George Washington University, Washington, DC 20037, USA

**Keywords:** sedentary behavior, physical inactivity, lifestyle activity, psychology, behavioral economics

## Abstract

The deleterious health effects of prolonged sitting and physical inactivity are well-established, yet these behaviors are pervasive in modern culture. To inform interventions aimed at reducing sedentary behavior and increasing lifestyle activity, this study examined psychological and behavioral economic factors that may be associated with these behaviors. This cross-sectional study was conducted among 4072 adults in Israel. Participants completed a survey pertaining to lifestyle behaviors and economic preferences using an online platform in September 2020. The psychological and behavioral economic factors of interest were patience, self-control, risk-taking, grit, and general self-efficacy. Sedentary behavior and lifestyle activity (e.g., time spent moving about) was assessed using the Rapid Assessment Disuse Index (RADI) tool (higher score indicative of more sitting and less activity). Multivariable linear and logistic regression analyses examined the association between psychological and behavioral economic factors and RADI score. Among 4072 participants, those who were impatient (vs. patient, β: −1.13; 95% CI: −1.89, −0.38) had higher grit (β: −1.25, 95% CI: −1.73, −0.77), and those who were more risk-seeking (β: −0.23; 95% CI: −0.33, −0.13) had lower RADI scores (i.e., less sedentary, more active). Significant associations for grit and risk-taking were also observed when the RADI score was dichotomized, such that individuals who had higher grit or were more risk-seeking were more likely to be non-sedentary/active. No significant associations were observed for self-control or general self-efficacy. Higher grit and more risk-seeking were associated with a decreased propensity for sedentary behaviors and inactivity; these factors may provide targets for interventions aimed at reducing sedentary behavior and increasing lifestyle activity.

## 1. Background

The deleterious health effects of physical inactivity and prolonged sitting (i.e., ‘disuse’ [1]) are well-established, but more than one-quarter (27.5%) of the global adult population is insufficiently active, including 36.8% of adults in high-income Western countries [2] and 30.7% of adults in Israel [3]. Total daily sitting time is also estimated to be the longest among adults in high-income countries (4.9 h) and has increased over time [4,5], related to more sedentary occupations, the growth in media technologies, and less active transport [6]. A large body of evidence attributes inactive lifestyles and sedentary behavior (i.e., activities in sitting/reclining positions while awake at an energy expenditure ≤1.5 metabolic equivalents) [7] to a range of adverse health outcomes, including type 2 diabetes, cardiovascular disease incidence and mortality, cancer incidence and mortality, and all-cause mortality [8,9]. Higher levels of physical activity have been shown to attenuate, though not always eliminate, associations between sedentary behavior and poor health outcomes [8]. 

To reduce sedentary behavior and increase activity at the population level, it is important to identify targetable factors that may influence these behaviors. One largely unexplored area is the influence of psychological and behavioral economic factors on an individual’s propensity toward sedentary behavior and lifestyle activity (e.g., time spent moving about during the day). Psychological and behavioral economic factors such as impatient time preferences (i.e., preferring immediate gratification over future rewards, or delay discounting) have been previously associated with adverse health behaviors including smoking and fast food consumption [10,11]. Impatient time preferences and inconsistent time preferences (e.g., self-control problems) are also related to lower physical activity levels [12,13,14].

However, little research has focused specifically on sedentary behavior and lifestyle activity, which are related to, but distinct from, physical activity [15]. Investigation of how time preferences and other psychological and behavioral economic factors relate to sedentary behavior and lifestyle activity may offer insight into more effective intervention design (e.g., short-term goal setting tailored to a participant’s time preferences, or using financial incentives for those with delay discounting tendencies [14]). For instance, time preferences have been targeted using financial incentives for smoking cessation and weight loss interventions [16,17], as well as for the promotion of physical activity [18,19], including among an Israeli population [20] 

We previously observed that sedentary behavior and inactivity were decreased among Israeli adults who experienced a COVID-19-related hardship, such as job loss. Within the same study population, we expand on that previous study to explore how several psychological and behavioral economic variables relate to sedentary time and lifestyle activity to inform the development and design of targeted interventions. 

## 2. Materials and Methods

Data for this study were derived from the Smoking and Lifestyles in Israel (SALI) study, which has been described previously [21]. Briefly, the SALI study was a cross-sectional survey conducted in September 2020 among a national sample of 4084 Israeli adults (ages 18–92 years); the study oversampled ever smokers to specifically examine tobacco use as one primary focus. Other areas of focus included in the survey were lifestyle behaviors, economic preferences, and psychological factors that influence decision making, which were the factors of interest in the present study. After providing informed consent, participants completed the survey on the online platform, iPanel, which adheres to the European Society for Opinion and Marketing Research standard and has approximately 100,000 active panel members across Israel [21,22,23]. In the current study, we explored the relationship of psychological and behavioral economic variables with sedentary behavior and lifestyle activity. Participants with incomplete information on any study covariate were excluded (n = 12), resulting in an analytic sample of 4072 participants. Each survey participant received 12.5 New Israeli Shekels (NIS), which was approximately USD 3.40 at the time of survey completion [21]. The University of Haifa Institutional Review Board (IRB) granted ethical approval for the overall study, and the study received exempt status from The Cooper Institute IRB. 

### 2.1. Predictors: Psychological and Behavioral Economic Factors

Time preferences (comprised of patience and self-control) were assessed using two survey questions that asked participants to make a decision based on hypothetical scenarios [11,24]. In the first question, participants chose between receiving a hypothetical monetary amount today or receiving a greater amount in 30 days. Three scenarios were provided, all of which included one option to receive 1000 NIS today or an alternative of: 1800 NIS in 30 days (scenario 1); 1500 NIS in 30 days (scenario 2); or 1200 NIS in 30 days (scenario 3). In the second question, participants chose between receiving a hypothetical monetary amount in 30 days or receiving a greater amount in 60 days. Again, three scenarios were provided, all of which included one option to receive 1000 NIS in 30 days or an alternative of: 1800 NIS in 60 days (scenario 1); 1500 NIS in 60 days (scenario 2); or 1200 NIS in 60 days (scenario 3). The two time preference parameters (patience and self-control) were derived from these survey questions, and calculated based on Laibson’s quasi-hyperbolic discount model [25], as previously described [24]. Participants were categorized as having patience, moderate patience, low patience, or impatience (patience variable), and as having present bias, future bias, or time consistent preferences (self-control variable).

Risk-taking was assessed using a one-item survey question [26]. Participants rated their risk-taking on a scale of 0–10, where 0 was not at all willing to take risks and 10 was very willing to take risks. Grit—or persistence and dedication to long-term goals even when faced with adversity—was assessed using a 10-item scale [27]. On a 5-point Likert scale, participants indicated the degree to which 10 statements (e.g., ‘Setbacks don’t discourage me. I don’t give up easily’) applied to themselves, from 1 (strongly disagree) to 5 (strongly agree). Points were summed and divided by 10 to yield a grit score ranging from 1 (not at all gritty) to 5 (extremely gritty). Finally, general self-efficacy—or one’s belief in their competence to successfully execute behaviors to meet the demands of a particular situation—was assessed using Chen’s New General Self-Efficacy Scale [28,29]. Participants indicated the degree to which eight statements (e.g., ‘When facing difficult tasks, I am certain that I will accomplish them’) applied to themselves, from 1 (strongly disagree) to 5 (strongly agree). Points were summed and divided by eight to yield a self-efficacy score ranging from 1 (least self-efficacy) to 5 (most self-efficacy). 

### 2.2. Outcome: Rapid Assessment Disuse Index (RADI) Score

The Rapid Assessment Disuse Index (RADI) was previously developed as a reliable and valid indicator of sedentary behavior and lifestyle activity [1]. The validation process, scoring protocol, and psychometric properties are described elsewhere [1]. Briefly, the RADI measured daily sitting (i.e., sedentary behavior), general moving about, and stair climbing (i.e., lifestyle activity parameters). Participants self-reported the frequency of each behavior (i.e., hours per day of sitting and moving about, and flights of stairs climbed) separately for the past week, month, and year. Scores for each behavior ranged from 1 to 5, with higher scores indicative of more sedentary behavior/inactivity. Scores were summed across each of the three behaviors and then across the three time periods. The overall RADI score ranges from 9 to 45 and is a summation of sitting and inactivity (i.e., ‘disuse’) over all time periods, with a higher score indicative of more disuse.

The RADI was analyzed as both a continuous outcome and a dichotomous outcome using a clinically relevant cut-point (<26 [non-sedentary] vs. ≥26 [sedentary]) [1]. This dichotomization had a sensitivity of 79% and a specificity of 63% compared to accelerometer-measured sedentary time among adults at a primary care clinic in the U.S [1]. Notably, the present study was conducted during a period of COVID-19-related school closures, curfews, and restricted travel in Israel; we previously showed that pandemic-related life events, particularly job loss, were related to less sedentary behavior in this study sample as measured by the RADI [21].

### 2.3. Statistical Analysis

Descriptive statistics were used to summarize participants’ characteristics. Various sociodemographic and health-related factors were identified as potential confounders a priori based on the prior literature and were adjusted for in multivariable models, including: age, gender (men/women/other), population group (Jewish/Arab/other), married (no/yes), college educated (no/yes), children aged <18 years in the household (no/yes), current smoking (no/yes), self-reported health status (poor/good/very good/excellent), and experiencing a major life event during COVID-19 (no/yes) [21]. Household income was also hypothesized to be a confounding factor but was not included in the primary multivariable models due to a high amount of missingness (~17%).

Multivariable regression models were used to examine the relationship between psychological/behavioral economic factors of interest and the RADI score. Linear regression was used to estimate beta (β) values and 95% confidence intervals (CIs) examining the RADI as a continuous outcome; heteroscedasticity-robust standard errors (SEs) were calculated to ensure consistent SEs even in the presence of heteroscedasticity [30]. Logistic regression was used to estimate odds ratios (ORs) and 95% CIs examining the RADI as a dichotomous outcome based on a clinically relevant cut-point defining non-sedentary/active (<26) and sedentary/inactive (≥26) [1]. We visually confirmed that residuals were symmetrically distributed and linearity assumptions were met. No multicollinearity issues were observed when examining variance inflation factors. To assess the effect of household income on observed associations, linear and logistic multivariable models were conducted among a subgroup of participants with nonmissing income data (n = 3371). Among this subgroup, estimates are presented with and without adjustment for income. 

Additionally, based on previous research [31], we explored potential effect modification by education; multivariable models were analyzed with an interaction term between college education and each of the predictors of interest in separate models with the continuous RADI score. Analyses were conducted in STATA SE V.17.0.

## 3. Results

Participant characteristics, as well as the distribution of the psychological/behavioral economic predictors and the RADI score, are provided in Table 1. Participants’ mean (standard deviation [SD]) age was 39.1 (14.2) years, with roughly half the sample being men (51.2%) and having a college education (45.8%). Most of the sample (87.1%) were Jewish, and 11.6% were Arab. Roughly one-third (29.4%) were current smokers, and most (72.0%) self-reported excellent or very good health. 

Nearly half of the participants (47.9%) had patient time preferences, and a majority (69.1%) were defined as time consistent (Table 1). Participants had a mean (SD) self-reported risk-taking score of 6.2 (2.2) (range 0–10, with higher scores indicative of more risk-taking); a mean (SD) grit score of 3.5 (0.5) (range 1–5, with higher scores indicative of more grit); and a mean (SD) general self-efficacy score of 4.0 (0.7) (range 1–5, with higher scores indicative of more self-efficacy). The mean (SD) RADI score was 27.9 (7.1) (range 9–45, with higher scores indicative of more sedentary/inactive behavior), and 63% of participants had a RADI score of at least 26—the clinical cut-point above which defined sedentary behavior/inactivity.

In multivariable linear regression analysis, those who were impatient (β: −1.13; 95% CI: −1.89, −0.38) had higher grit scores (β: −1.25, 95% CI: −1.73, −0.77), and those who were more risk-seeking (β: −0.23; 95% CI: −0.33, −0.13) had significantly lower RADI scores, meaning they sat less and moved more (Table 2). Self-control and general self-efficacy were not associated with RADI score. In multivariable logistic regression analysis, impatience was no longer significantly associated with less sedentary behavior/inactivity (i.e., a low RADI score of <26), although grit and risk-taking remained statistically significant (Table 3). That is, participants with higher grit scores (OR: 1.42, 95% CI: 1.23, 1.65) or more risk-taking preferences (OR: 1.05, 95% CI: 1.02, 1.09) were more likely to have a low RADI score and be defined as non-sedentary/active. No significant associations were observed between self-control or self-efficacy and the dichotomized RADI score. Including household income in the analysis did not substantively change the results (Table 2 and Table 3). Additionally, no significant interactions were observed when assessing effect modification of the association between psychological/behavioral economic factors and continuous RADI score by education.

## 4. Discussion 

Among a large sample of Israeli adults, impatience, higher grit, and more risk-taking were independently associated with less sitting time and more moving about. Other psychological and behavioral economic factors examined—self-control and general self-efficacy—were not associated with sedentary behavior or inactivity. These data offer a novel, preliminary insight into largely unexplored psychological and behavioral economic factors that could be targeted in interventions designed to reduce sitting time and replace it with lifestyle activity. Sedentary behavior has been a focus of recent intervention work due to its significant relationship with chronic diseases and premature mortality [32,33]. Given the World Health Organization’s recommendation to limit sedentary time and replace it with physical activity at any intensity level [15], replacing sitting time with light intensity lifestyle activity (e.g., casual walking, housework) might be a more achievable goal than engaging in moderate to vigorous intensity activity for adults who are currently inactive [34].

Present findings revealed that impatience—difficulty delaying immediately gratifying behavior for higher-level goals [35]—was associated with decreased sedentary behavior/inactivity, though the association was small and not observed when the RADI score was dichotomized. Conversely, previous studies have found that impatience is associated with less physical activity [13,36,37]. This difference may be driven by the fact that sedentary time and physical activity are distinct behaviors [38]. When comparing sitting to leisure-time physical activity, for instance, the latter could be more intentional and planned than the former [39]. Specifically, physical activity—which requires goal setting and planning—might be less impacted by non-conscious factors (e.g., environmental cues activating behavior) than sedentary behavior [40,41]. This hypothesis, however, cannot be confirmed in the present study since we did not measure implicit attitudes nor leisure-time activity. 

Additionally, study findings reveal that risk-seeking preferences are associated with lower RADI scores; namely, with less sitting and more moving about activity. To our knowledge, no prior research has specifically examined risk preferences and sitting time. However, a few studies have examined the relationship between risk-taking and physical activity, which have found mixed results. For example, van der Pol et al. did not observe a relationship between risk-taking and adherence to doctors’ advice on physical activity (and diet) [42], whereas Leonard et al. reported a significant association between risk-seeking preferences and a higher physical activity stage of change [37]. The current results are consistent with the latter study, though the focus in the current study is on sedentary behavior and lifestyle activity, rather than leisure-time physical activity specifically. In addition, our findings pertaining to grit—persistence and passion towards long-term goals [27]—demonstrate that it is associated with a low RADI score (less sedentary, more moving about). This aligns with prior studies observing a relationship between grit, physical activity, and sitting time [43], including less sedentary time and more activity during the COVID-19 pandemic [44]. Research on interventions that effectively increase grit, particularly among young people, remains sparse, and these warrant further investigation [45]. 

Several limitations should be noted. Temporality and causality cannot be determined given the cross-sectional study design. The assessment of psychological and behavioral economic factors at a single point limits our understanding of how these factors may vary over time to influence lifestyle activity and sedentary behaviors. The study sample is not representative of the Israeli population at large. Finally, the study was conducted during the COVID-19 pandemic, which we have previously shown was associated with changes in sitting behavior and lifestyle activity [21]; this time period may not be reflective of attitudes and behaviors prior to or after the pandemic. 

## 5. Conclusions

This study adds to the limited literature on the psychological and behavioral economic predictors of sedentary behavior and lifestyle activity among adults. Data from this large sample in Israel offer preliminary insights into novel factors that can be utilized when designing interventions to reduce sedentary time and physical inactivity while aiming to replace this behavior with lifestyle activity (e.g., housework, taking the stairs). Future longitudinal research among diverse samples, including other countries, should further explore how psychological and behavioral economic factors such as patience, grit, and risk-seeking affect sedentary behavior and inactivity and subsequently reduce chronic disease and improve overall public health. 

## Figures and Tables

**Table 1:** Characteristics of participants in the Smoking and Lifestyles in Israel study, September 2020 (n = 4072).

Characteristic	n (%)
Time preferences: patience	
Impatient	530 (13.0)
Low patience	330 (8.1)
Moderate patience	1263 (31.0)
Patient	1949 (47.9)
Time preferences: self-control	
Present bias	278 (6.8)
Time consistent	2812 (69.1)
Future bias	982 (24.1)
Gender	
Men	2083 (51.2)
Women	1985 (48.8)
Other	4 (0.1)
Population group	
Jewish	3548 (87.1)
Arab	471 (11.6)
Other	53 (1.3)
Marital status	
Married or partnered	2607 (64.0)
Not married	1465 (36.0)
College education	
Yes	1866 (45.8)
No	2206 (54.2)
Monthly household income, NIS ^a^	
≤4000	244 (7.2)
4001–8000	485 (14.4)
8001–12,000	847 (25.1)
12,001–17,000	910 (27.0)
17,001–22,000	555 (16.5)
>22,000	330 (9.8)
Missing	701
Children < 18 years in household	
Yes	1747 (42.9)
No	2325 (57.1)
Current smoker	
Yes	1198 (29.4)
No	2874 (70.6)
Self-reported health status	
Excellent	1250 (30.7)
Very good	1682 (41.3)
Good	940 (23.1)
Poor	200 (4.9)
Major change or hardship from COVID-19	
Yes	1763 (43.3)
No	2309 (56.7)
	Mean (SD)
Age (years)	39.1 (14.2)
Risk-taking (range: 0–10) [higher = more risk-taking]	6.2 (2.2)
Grit (range: 1–5) [higher = grittier]	3.5 (0.5)
General self-efficacy (range: 1–5) [higher = more self-efficacy]	4.0 (0.7)
RADI score (range: 9–45) [higher = more sedentary]	27.9 (7.1)

Abbreviations: NIS, New Israeli Shekels; RADI, Rapid Assessment Disuse Index; SD, standard deviation; ^a^ percentages exclude missing values.

**Table 2:** Association between psychological/behavioral economic factors and continuous RADI score ^a^ among study participants in the Smoking and Lifestyles in Israel study.

	Full Sample (n = 4072)	Subgroup with Nonmissing Income (n = 3371)	
		No Income Adjustment	Income Adjustment
	Adjusted β (95% CI) ^b^	Adjusted β (95% CI) ^b^	Adjusted β (95% CI) ^c^
Time preferences: patience (referent: patient)			
Impatient	−1.13 (−1.89, −0.38)	−1.27 (−2.10, −0.45)	−1.12 (−1.94, −0.30)
Low patience	−0.29 (−1.19, 0.60)	0.15 (−0.83, 1.13)	0.21 (−0.77, 1.18)
Moderate patience	−0.27 (−0.81, 0.27)	0.05 (−0.54, 0.64)	0.06 (−0.53, 0.65)
Time preferences: self-control (referent: future bias)			
Present bias	−0.60 (−1.53, 0.32)	−0.73 (−1.74, 0.28)	−0.67 (−1.68, 0.34)
Time consistent	−0.21 (−0.81, 0.39)	−0.21 (−0.86, 0.45)	−0.23 (−0.88, 0.43)
Risk-taking (higher = more risk-taking)	−0.23 (−0.33, −0.13)	−0.21 (−0.32, −0.10)	−0.23 (−0.34, −0.12)
Grit (higher = grittier)	−1.25 (−1.73, −0.77)	−1.18 (−1.70, −0.65)	−1.21 (−1.73, −0.69)
General self-efficacy (higher = more self-efficacy)	−0.12 (−0.52, 0.27)	−0.15 (−0.59, 0.29)	−0.22 (−0.65, 0.22)

Abbreviations: CI, confidence interval; RADI, Rapid Assessment Disuse Index; ^a^ higher RADI scores indicate increased sedentary behavior and inactivity. Conversely, lower scores indicate less sitting and more moving about; ^b^ model adjusted for age, gender, population group, marital status, college education, children < 18 years living in the household, self-reported health status, and experiencing a major change or financial hardship due to COVID-19; ^c^ model adjusted for age, gender, population group, marital status, college education, children < 18 years living in the household, self-reported health status, experiencing a major change or financial hardship due to COVID-19, and monthly household income.

**Table 3:** Association between psychological/behavioral economic factors and non-sedentary behavior (RADI score < 26) among study participants in the Smoking and Lifestyles in Israel study.

	Full Sample (n = 4072)	Subgroup with Nonmissing Income (n = 3371)	
		No Income Adjustment	Income Adjustment
	Adjusted OR (95% CI) ^a^	Adjusted OR (95% CI) ^a^	Adjusted OR (95% CI) ^b^
Time preferences: patience (referent: patient)			
Impatient	1.23 (0.98, 1.55)	1.27 (0.99, 1.63)	1.24 (0.96, 1.59)
Low patience	1.05 (0.79, 1.40)	0.92 (0.67, 1.26)	0.91 (0.66, 1.25)
Moderate patience	1.00 (0.85, 1.19)	0.90 (0.75, 1.09)	0.90 (0.74, 1.08)
Time preferences: self-control (referent: future bias)			
Present bias	1.22 (0.90, 1.64)	1.24 (0.89, 1.72)	1.23 (0.88, 1.71)
Time consistent	1.01 (0.84, 1.23)	1.02 (0.83, 1.26)	1.03 (0.84, 1.28)
Risk-taking (higher = more risk-taking)	1.05 (1.02, 1.09)	1.05 (1.02, 1.09)	1.05 (1.02, 1.09)
Grit (higher = grittier)	1.42 (1.23, 1.65)	1.35 (1.15, 1.59)	1.37 (1.16, 1.61)
General self-efficacy (higher = more self-efficacy)	1.05 (0.93, 1.18)	1.06 (0.93, 1.22)	1.08 (0.94, 1.23)

Abbreviations: CI, confidence interval; OR, odds ratio; RADI, Rapid Assessment Disuse Index; ^a^ model adjusted for age, gender, population group, marital status, college education, children < 18 years living in the household, self-reported health status, and experiencing a major change or financial hardship due to COVID-19; ^b^ model adjusted for age, gender, population group, marital status, college education, children < 18 years living in the household, self-reported health status, experiencing a major change or financial hardship due to COVID-19, and monthly household income.

## Data Availability

SALI study data are not publicly available. A scientific data request may be submitted to The Cooper Institute’s Scientific Review Board Committee for review.
